# Forecasting COVID-19 Epidemic Trends by Combining a Neural Network with *R_t_* Estimation

**DOI:** 10.3390/e24070929

**Published:** 2022-07-04

**Authors:** Pietro Cinaglia, Mario Cannataro

**Affiliations:** 1 Department of Health Sciences, Magna Graecia University of Catanzaro, 88100 Catanzaro, Italy; 2 Department of Medical and Surgical Sciences, Magna Graecia University of Catanzaro, 88100 Catanzaro, Italy; cannataro@unicz.it

**Keywords:** time series, deep learning, neural networks, COVID-19, SARS-CoV-2, epidemic trend

## Abstract

On 31 December 2019, a cluster of pneumonia cases of unknown etiology was reported in Wuhan (China). The cases were declared to be Coronavirus Disease 2019 (COVID-19) by the World Health Organization (WHO). COVID-19 has been defined as SARS Coronavirus 2 (SARS-CoV-2). Some countries, e.g., Italy, France, and the United Kingdom (UK), have been subjected to frequent restrictions for preventing the spread of infection, contrary to other ones, e.g., the United States of America (USA) and Sweden. The restrictions afflicted the evolution of trends with several perturbations that destabilized its normal evolution. Globally, $R_{t}$ has been used to estimate time-varying reproduction numbers during epidemics. *Methods:* This paper presents a solution based on Deep Learning (DL) for the analysis and forecasting of epidemic trends in new positive cases of SARS-CoV-2 (COVID-19). It combined a neural network (NN) and an $R_{t}$ estimation by adjusting the data produced by the output layer of the NN on the related $R_{t}$ estimation. *Results:* Tests were performed on datasets related to the following countries: Italy, the USA, France, the UK, and Sweden. Positive case registration was retrieved between 24 February 2020 and 11 January 2022. Tests performed on the Italian dataset showed that our solution reduced the Mean Absolute Percentage Error (MAPE) by 28.44%, 39.36%, 22.96%, 17.93%, 28.10%, and 24.50% compared to other ones with the same configuration but that were based on the LSTM, GRU, RNN, ARIMA (1,0,3), and ARIMA (7,2,4) models, or an NN without applying the $R_{t}$ as a corrective index. It also reduced MAPE by 17.93%, the Mean Absolute Error (MAE) by 34.37%, and the Root Mean Square Error (RMSE) by 43.76% compared to the same model without the adjustment performed by the $R_{t}$. Furthermore, it allowed an average MAPE reduction of 5.37%, 63.10%, 17.84%, and 14.91% on the datasets related to the USA, France, the UK, and Sweden, respectively.

## 1. Introduction

On December 31, 2019, a cluster of pneumonia cases of unknown etiology was reported in Wuhan (China), which was declared to be Coronavirus Disease 2019 (COVID-19) by the World Health Organization (WHO). COVID-19 rapidly spread worldwide, degenerating from an epidemic to a pandemic on 11 March 2020 [1]. COVID-19 principally involves the respiratory tract, and the clinical presentation is very similar to that of Severe Acute Respiratory Syndrome (SARS), which there was an outbreak of in 2003. According to this similarity, COVID-19 has been defined as SARS Coronavirus 2 (SARS-CoV-2).

Infectious disease outbreaks exhibit a set of patterns that may be identified by studying their transmission dynamics [2]. COVID-19 showed a dynamic transmission during the epidemic on specific patterns that depended on several aspects, such as density of population, infection rate, ratio of the number of vaccinated people to the number of non-vaccinated people, and lifestyle [3]. In epidemiology, these aspects are proper to each state, country, or region, thus a mathematical model designed for a specific area cannot fit optimally with others [4], the same observation applies between a sub-area and its super-area [5].

As with other epidemics [6,7], data for COVID-19 were made available as a series of data points indexed in time order (i.e., as time-series) [8].

This paper presents a solution based on deep learning (DL) for the analysis and forecasting of epidemic trends in new positive cases of SARS-CoV-2 (COVID-19). It combined a neural network (NN) and $R_{t}$ estimation by adjusting the data produced by the output layer of the NN on the related $R_{t}$ estimation. The latter allowed the estimation of time-varying reproduction numbers during the epidemic, its value was computed for each prediction in order to adjust the final forecasting.

The NN was chosen on the basis of tests performed on the assumption described below. Generally, NNs are not suitable for historical data with respect to the RNN. However, the absence of long-term memory might be an advantage in the case in which unforeseeable external actions can occur within the time series, which can frequently destabilize the long short-term memory, e.g., lockdowns, and/or limitations. To give an example, Italy was subjected to emergency improvements that introduced restrictions for preventing the spread of the infection [9,10]. The proposed solution was focused on the Italian dataset in order to provide an effective model to handle what has been described. In addition, we performed tests on the following other countries to demonstrate its evolvability and reusability: the United States of America (USA), France, the United Kingdom (UK), and Sweden. Italy, France, and the UK were subjected to frequent restrictions for preventing the spread of the infection, contrary to the USA and Sweden.

In addition, our solution was able to perform several analyses that were useful for studying the trend related to positive cases. For instance, it reported statistical information related to the descriptive analysis, as well as the related plots. Our solution, compared with other models based on DL, exhibited greater accuracy and robustness to variations.

The rest of the paper is organized as follows. Section 3 reports details about the datasets used for training and analysis, model design, and data processing. Section 4 shows a set of tests that were performed to validate the proposed solution. Section 5 reports a discussion about the results shown in Section 4, and it highlights the objectives achieved according to accuracy and robustness. Furthermore, a comparison with other methodologies is discussed to corroborate the chosen network implemented within our solution.

## 2. Background

Time-series data are sequences of numerical values that have associated time stamps. The trend of a time series may be studied through two main models: (i) statistical models, or (ii) machine learning (ML) models. Usually, the former is performed by implementing the autoregressive integrated moving average (ARIMA) [11].

The ARIMA is a generalization of the autoregressive moving average (ARMA) [12] that includes the autoregressive (AR) model and the moving average (MA) model. ARIMA is based on three processes: auto-regression, integration, and moving average [13]. Furthermore, seasonal ARIMA (SARIMA) [14] is an extension of the ARIMA, supporting the modeling of a seasonal component in the series. A SARIMA model is built by including additional seasonal terms in the ARIMA: the seasonal parts of the model are the terms that involve back shifts in the seasonal period.

The ARIMA and SARIMA models have been applied in several studies for predicting the spread of an epidemic [15], COVID-19 included [16,17], as well as to estimate the fatality rate [18].

The statistical models required several assumptions, e.g., the starting point of the infectious disease, the interactions between people (related to incubation period), as well as the input parameters for the model. Usually, the input parameters needed to be estimated through a deductive approach based on repeated tests [19]. For instance, an ARIMA model needs an underlying process that generates the observations (i.e., ARIMA process), in order to estimate the following parameters [20]:

- *lag order (p)*: the number of lag observations included in the model;
- *degree of differencing (d)*: the number of times that the raw observations are differenced;
- *order of moving average (q)*: the size of the moving average window.

ArunKumar et al. [21] investigated the dynamics of cumulative COVID-19 cases in 16 countries (South Africa, Bangladesh, Brazil, Chile, Columbia, India, Iran, Italy, Mexico, Pakistan, Peru, Russia, Saudi Arabia, Spain, the UK, the USA) by using ARIMA models based on forecasting the cumulative analysis to estimate the best ARIMA (p,d,q) for each country. To give an example, the authors indicated that ARIMA (7,2,4) and ARIMA (3,1,6) were the best models for confirmed cases and death cases in Italy, respectively. Results also indicated that COVID-19 trends could be classified into three main classes: exponential rise (the USA, South Africa, Colombia, Brazil, India, Mexico, and Bangladesh), steep linear increment (Saudi Arabia, Pakistan, Chile, Russia, Peru, Iran), and gradual linear increment (Italy, the UK, and Spain).

As mentioned, a second method for studying the trend of a time series is based on ML [22], and more appropriately on a class of ML called DL [23]. These allow the assumptions required for statistical approaches to be overcome [24]. DL models have proven to be an effective technique for time series analysis, including the forecasting of an outbreak of COVID-19 [25]. In DL, an NN (or circuit of neurons) is used for solving an artificial intelligence (AI) issue. The latter has been explored in almost every field of medicine [26,27,28]. DL techniques have a significant accuracy rate both for the spread and dynamics of the COVID-19 epidemic, and for its early detection [29].

Usually, an NN is used to process both sequential and temporal data (e.g., time series) [30], and it can be integrated with a long short-term memory (LSTM) to support a gated memory unit that is able to handle the vanishing gradient problems. LSTM is a recurrent network architecture combined with a gradient-based learning algorithm to overcome back-flow problems [31]. LSTM uses its memory cells to remember long-range information and track the various attributes of text it is currently processing [32]. Chimmula et al. [33] analyzed data collected from Johns Hopkins University and the Canadian Health authority by developing a DL approach based on LSTM [32].

Ayoobi et al. [34] examined six different DL methods on COVID-19 data for Australia and Iran to investigate time series forecasting of new cases and the rate of new deaths for COVID-19. In detail, the study performed an in-depth comparison of LSTM, convolutional LSTM (Conv-LSTM) [35], and gated recurrent units (GRUs) [36] along with their bidirectional extensions: bidirectional-LSTM (Bi-LSTM), bidirectional convolution LSTM (Bi-Conv-LSTM), and bidirectional GRU (Bi-GRU), respectively [37]. The comparison performed by Ayoobi et al. produced the following key observations: Conv-LSTM, Bi-Conv-LSTM, and GRU, obtained the best rank for the prediction of new cases over 1, 3, and 7 days, respectively, for the Australian dataset. Conv-LSTM obtained the best rank for new deaths in all tests for the Australian dataset (i.e., prediction over 1, 3, and 7 days). For the Iranian dataset, results were even more heterogeneous. Therefore, the mentioned study suggested that the best DL model did not exist for any dataset, but that they must be assessed on a case-by-case basis.

Similarly, Zeroual et al. [38] investigated DL methods focusing on NNs, and recurrent NNs (RNNs). In an NN, the data flow transformations can be passed via hidden layers in one direction by producing an output that is affected only by the current situation. Otherwise, an RNN has a low memory that may be increased by including an LSTM hidden layer or a GRU.

NNs are usually employed to study time series forecasting models. Time series are a crucial issue for several topics, including epidemiology [39].

Let us denote the current time stamp with $h_{t}$, the time stamp at previous state with $h_{t-1}$, the weight matrix with *W*, the bias matrix with *b*, and the hyperbolic tan function with tanh. Formally, the function of an NN cell may be represented by using the following equation:

$$h_{t}=tanh\left(W\left[h_{t-1},x_{t}\right]+b\right)$$

Briefly, each cell calculates $h_{t}$ by performing a hyperbolic tan function in which $h_{t-1}$ and $x_{t}$ are combined with a weight matrix, adding also a specific bias. The hyperbolic tan function allows scaling the actual values within the range [−1,+1]. Therefore, the output of a cell is updated by using the following sigmoid function (f(x)) at each time step:

$$f\left(x\right)=1/\left(1+e^{-x}\right)$$

**Figure 1:**
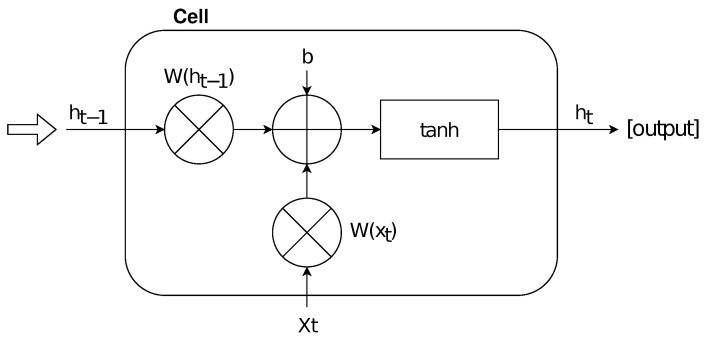
The schematic representation of a single NN cell.

According to the equations above, Figure 1 shows the schematic representation for an NN cell; note that the output produced by a cell can be imported as input for a subsequent one.

An NN recollects only recent information, unless its memory is extended, e.g., with an LSTM or GRU. As discussed, NN are not suitable for historical data in respect to LSTM. However, external actions could occur within the time series, destabilizing the long short-term memory, e.g., lockdowns, and/or limitations. To give an example, Italy was subjected to emergency improvements that introduced restrictions for preventing the spread of COVID-19. For instance, our tests demonstrated that LSTM for our dataset was counterproductive, contrary to the datasets of countries where government interventions were less frequent. To give an example, Ma et al. [40] analyzed epidemic trends in the US, the UK, Brazil, and Russia by combining LSTM with the Markov method to optimize the results, because the pure use of LSTM had proven to be not optimal. In Chandraa et al. [41], an approach based on LSTM resulted in data that was highly biased by a single major peak, when the entirety of India’s cases were considered.

## 3. Materials and Methods

This section presents the methodology that was applied for designing the proposed solution. This one was based on an in-house algorithm that implemented a model based on the NN in order to support a time-dependent model with event persistence. Furthermore, we adjusted the data produced by the output layer of the NN by using the $R_{t}$ estimation as a corrective index. As described, $R_{t}$ allows the estimation of time-varying reproduction numbers during epidemics, and its value was computed for each prediction in order to adjust the final forecasting. It was integrated downstream of the output layer of the NN.

**Figure 2:**
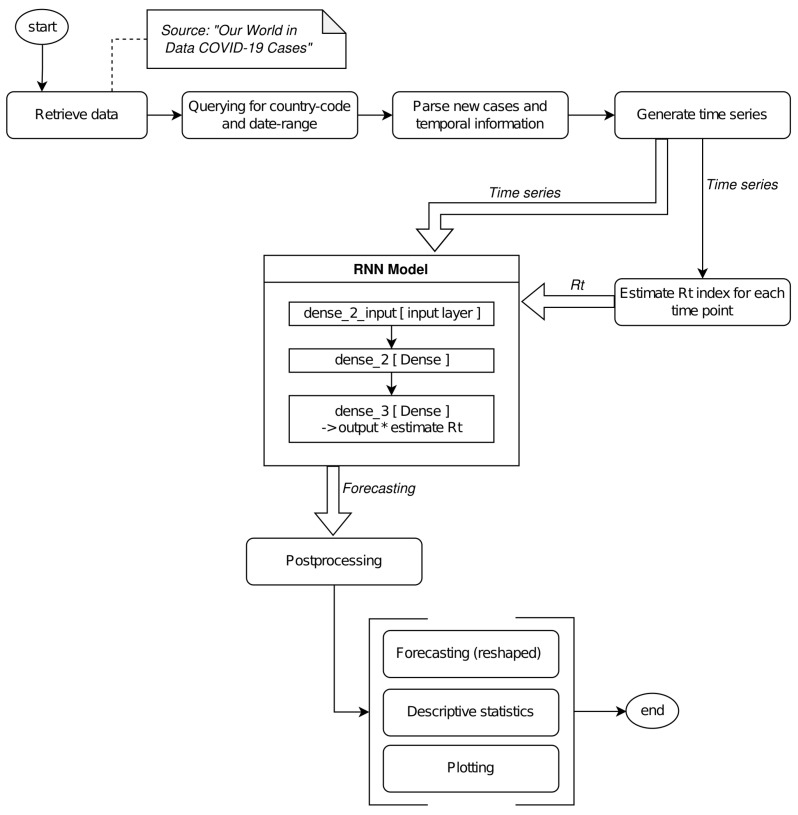
Figure reports the pipeline for our solution. We parsed data by retrieving the positive case registration as a time series.

The proposed solution was based on the following main steps: (i) pre-processing, (ii) NN modeling, (iii) prediction, (iv) post-processing. In preprocessing, the data were parsed by filtering only the information of interest and were reshaped into the [*samples, time steps, features*] format. The NN was modeled by integrating the $R_{t}$ estimation during its computation. Finally, data were subject to an inverse transformation according to the original time-series. The pipeline is shown in Figure 2.

### 3.1. Dataset

The proposed solution retrieved data from the “Our World in Data COVID-19 Cases” dataset (ourworldindata.org/covid-cases, accessed on 11 January 2022) [42]. This dataset is a freely available resource under a Creative Commons license that aggregates datasets provided by government agencies and research institutes for 207 countries. For instance, the Italian dataset was acquired by the official GitHub Repository provided by the Department of Italian Civil Protection. It includes daily information related to the tracking of COVID-19 trends, such as tested confirmed cases, the number of people who reportedly died, and the number of people who reportedly recovered from COVID-19.

We focused our attention on Italy, extending the analysis to the USA, France, the UK, and Sweden, in order to demonstrate the evolvability and reusability of our solution. The data sources aggregated in the “Our World in Data COVID-19 Cases” dataset for the listed countries are reported in Table 1.

Our algorithm only used information related to new positive cases (or new cases) for computation and forecasting, therefore other ones were excluded. New cases consisted of the difference between the total number of cases on the current day and the total number of cases from the previous day. This information could be used to construct a time series related to the daily change in new cases. New case registration was retrieved between 24 February 2020 and 11 January 2022; Table 2 reports the descriptive statistics both for new cases and total cases.

Furthermore, we investigated the effective reproduction number of new cases (i.e., $R_{t}$) [43] by applying the method presented by Huisman et al. [44]. The latter is based on the approach proposed by Cori et al. [45] to estimate time-varying reproduction numbers during epidemics.

**Figure 3:**
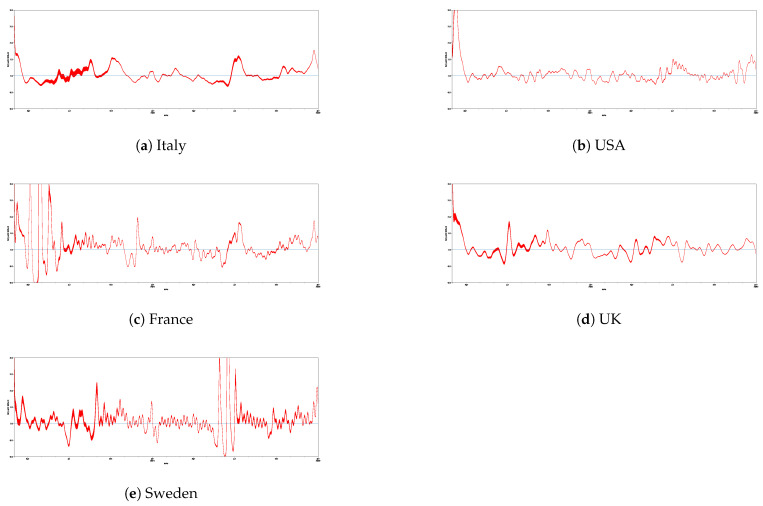
The trends related to the $R_{t}$ estimation for Italy, the USA, France, the UK, and Sweden.

Table 3 reports the descriptive statistics related to the study of the $R_{t}$ index for the examined countries (i.e., Italy, the USA, France, the UK, and Sweden). Figure 3 shows the trend related to $R_{t}$ estimation for Italy, the USA, France, the UK, and Sweden.

### 3.2. Preprocessing

This step retrieved the data of interest in comma-separated value (CSV) format. A country can be indicated by its country code, according to the related International Standards Organization (ISO) 3166-1 standard. For instance, the dataset provided by the Department of Italian Civil Protection can be retrieved from the “Our World in Data COVID-19 Cases” dataset by using “ITA” as the ISO code, similarly the dataset for the United State of America can be retrieved by using “USA”. Furthermore, a date range can be defined to extract only a subset of the information.

Each dataset contains information that is not relevant to our model. The preprocessing step extracted the information of interest, which consisted of new positive cases.

**Figure 4:**
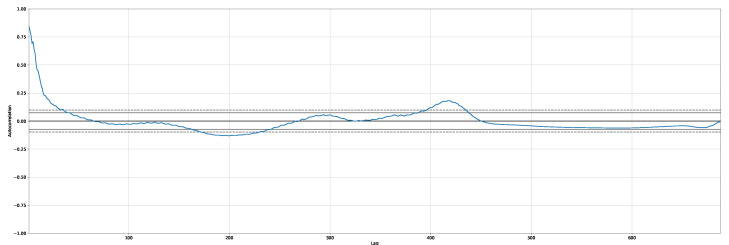
An example of autocorrelation plot for new positive cases. Dataset: Italy.

We studied stationary points for the time-series by analyzing the autocorrelation. To give an example, Figure 4 shows the autocorrelation plot for the new positive cases related to the Italian dataset.

To build the training and testing datasets, we defined a function based on two main arguments: (i) the original dataset and (ii) the number of previous time steps to use as input variables to predict the next time period (i.e., look_back). This default created a dataset where *X* was the number of observations at a given time (*t*) and *Y* was the number of observations at the next time (t+look_back). We used a look_back value of 7 (7 days or 1 week) for training.

To build the NN model, data were subject to a transformation. The resulting format was [*samples, time steps, features*]. The samples consisted of information from the previous days according to look_back; the time step was 1 day (data were collected daily), and the features were the new positive cases.

We split the time-series into two sets: training and testing. The split point was fixed to 80% of the observations for training, and the remaining 20% were for testing. After this first splitting, we kept aside the test set and we randomly chose 80% of the training set as the new training set, and the remaining (20%) was the validation set.

### 3.3. Model

According to several studies for the prediction of short-term stock price trends [46,47,48,49], we built a DL method based on a very-short-term time-series forecaster [50].

In Ma et al. [40], epidemic trends in the US, Britain, Brazil, and Russia were analyzed by combining LSTM with the Markov method to optimize the result, because the pure use of LSTM has been proven to be not optimal. In Chandraa et al. [41], an approach based on LSTM was highly biased by a single major peak, when the entirety of India’s cases were considered.

The proposed NN was based on two main layers: (i) a standard fully-connected (dense) layer with a rectified linear unit (ReLU) activation and (ii) a custom dense output layer.

ReLu activation is defined as the positive part of its argument:

$$f\left(x\right)=x^{+}=\max\left(0,x\right)$$

A major benefit of ReLu is that the gradient has a constant value, therefore it allows faster learning.

The configuration for our NN consisted of four neurons for the dense layer, and one neuron for the output layer. Furthermore, it used the Adam [51] for first-order gradient-based optimization of stochastic objective functions.

The proposed solution was affected by a correction based on $R_{t}$ estimation, in order to also consider the time-varying reproduction numbers during epidemics. The estimation of *R* at time *t* (i.e., $R_{t}$) requires incidence data. Let us denote $w_{s}$ as the probability distribution, which is dependent on the time since infection of the case *s*, and independent of time *t*. Let $I_{t}$ be the ratio of the number of new positive cases at time *t* and $I_{t-s}$ be the incidence at t−s. The $R_{t}$ is formally defined as follows:

$$R_{t}=\sum_{s=1}^{t}I_{t-s}w_{s}$$

According to this equation, the incidence of cases at time *t* may be calculated as follows:

$$E\left[I_{t}\right]=R_{t}\sum_{s=1}^{t}I_{t-s}w_{s}$$

$E[I_{t}]$ denotes the expectation at $I_{t}$.

Briefly, $R_{t}$ is an index reflecting the incident rate: the higher $R_{t}$ is the higher the growth rate of infection is; therefore, a higher infection is expected in the next period. The computation was based on the formula described above. Our own in-house function computed the $R_{t}$ index for each forecasting by weighting the latter on the same $R_{t}$ index, as a multiplicative factor. More implementation details are reported in Section 3.5

### 3.4. Postprocessing

This step had the sole purpose of returning the data into their original format. Therefore, data predicted by NN should be inverse-transformed starting from the dataset obtained by pre-processing (i.e., [*samples, time steps, features*]), into the time-series format (i.e., [*date, new positive cases*]). The inverse transformation allowed both the descriptive analysis of the predicted trend and the plotting.

### 3.5. Implementation

The proposed solution was implemented in Python (version 3) [52], by using the following set of well-known packages:

- *pandas* [53]. This package is a software library for data analysis and manipulation. It includes a data structure to handle data frames efficiently. Furthermore, time-series are supported; for instance, it allows date range generation and frequency conversion, statistics, date shifting, and lagging.
- *numpy* [54]. This package provides support for large, multidimensional arrays and matrices, as well as a collection of high-level mathematical functions to manipulate these data structures.
- *tensorflow* [55]. This package is one of the most widely used end-to-end open source platforms for ML/DL.
- *scikit-learn* [56]. This package is a collection of tools for predictive data analysis, classification, regressions, and clustering. It supports the interoperability with other packages (e.g., numpy).
- *epyestim* [45]. This package is able to estimate time-varying reproduction numbers (i.e., $R_{t}$) from epidemic curves. It is provided in both software tool and package form. The latter also supports the Python languages.

The proposed solution used *numpy* and *pandas* to handle the data, while the NN was modeled through *tensorflow*. Furthermore, the $R_{t}$ estimation was computed by using *epyestim* and was included within the output layer. Therefore, the contagion rate estimated by $R_{t}$ afflicted the original output by weighting it. From a pure implementation point of view, we used the *custom Dense layer* of *tensorflow* to include our own function. The latter worked as a multiplying factor by weighting the original output on the estimated $R_{t}$: $originalOutput*estimatedR_{t}$.

We implemented this solution on Google Colaboratory (Colab) [57], which we also used as a test environment. Colab is a research project for prototyping ML/DL models on Google Compute Engine (GCE) by ensuring powerful hardware options (e.g., GPUs and TPUs). GCE is the infrastructure as a service (IaaS) component of the Google Cloud Platform.

### 3.6. Key Performance Indicators

According to comparative studies of ML/DL methods for time-series forecasting [58,59], the proposed model was evaluated based on three well known key performance indicators (KPIs): Mean Absolute Error (MAE) [60], Mean Absolute Percentage Error (MAPE) [61], and Root Mean Square Error (RMSE) [62]. MAE, MAPE, and RMSE are the most common metrics that are widely adopted to measure forecasting accuracy, as well as to evaluate both NN and statistical models.

These are defined as follows:

$$MAE=\frac{1}{n}\sum_{i=1}^{n}\left|y_{i}-{\hat{y}}_{i}\right|$$

$$MAPE=\frac{1}{n}\sum \frac{\left|y_{i}-{\hat{y}}_{i}\right|}{d_{t}}$$

$$RMSE=\sqrt{\frac{1}{n}\sum_{i=1}^{n}{\left(y_{i}-{\hat{y}}_{i}\right)}^{2}}$$

Within the equations reported above, $y_{i}$ and ${\hat{y}}_{i}$ are the observed and predicted data, respectively, while *n* is the size of the observed data.

There is no one-size-fits-all indicator to evaluate forecast accuracy (or error) [63].

MAPE is the sum of the individual absolute errors divided by each period (i.e., demand), separately. It represents the average of the percentage errors, and it expresses model prediction error in terms of MAE percentage. According to its definition, MAPE is a really strange forecast KPI for comparison. As neither MAE nor RMSE are scaled to the demand, these need to be normalized to the average demand before they can evaluate if their resulting value is good or bad. This feature makes them a particularly good KPI to evaluate a single model, but not for comparison between different ones.

## 4. Results

Tests were performed in a standard Colab environment, which had the following configuration:

- Type: n1-highmem-2 instance;
- CPU: 2vCPU @ 2.2 GHz;
- RAM: 13 GB;
- Backend: GCE, Python 3.

Benvenuto et al. [64] reported ARIMA (1,0,3) as the best ARIMA model for determining the incidence of COVID-19. However, according to [21], ARIMA (7,2,4) is the best model for confirmed cases. We used both ARIMA models for our tests.

According to what was discussed in Section 3.6, results were evaluated on the MAPE indicator to compare the proposed NN and other solutions available in the literature. MAPE was oriented negatively (lower was better). However, RMSE and MAE were reported, for completeness.

**Figure 5:**
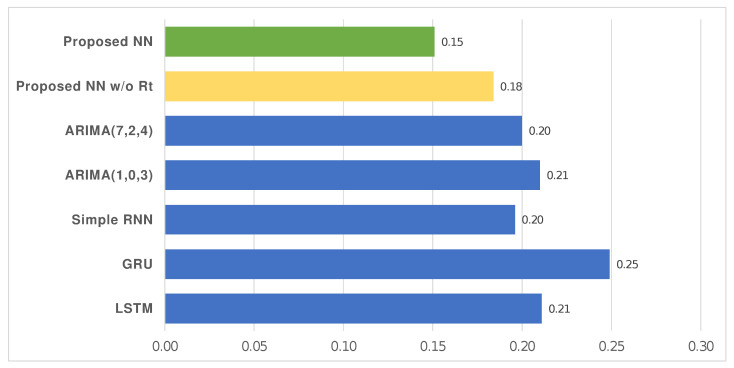
MAPE for the proposed model with and without the correction computed by using the $R_{t}$ estimation (i.e., proposed NN and proposed NN without $R_{t}$, respectively), as well as for the other models based on DL (i.e., LSTM, Simple RNN, GRU) and ARIMA. MAPE was oriented negatively (lower was better). Dataset: Italy.

Figure 5 shows the MAPE calculated for the proposed model on the Italian dataset, as well the following ones used for comparison: LSTM, GRU, Simple RNN, ARIMA. More specifically, the forecasting was performed multiple times (N=10) for each model by reporting the resulting average value for the MAPE; the related descriptive statistics are shown in Table 4.

**Figure 6:**
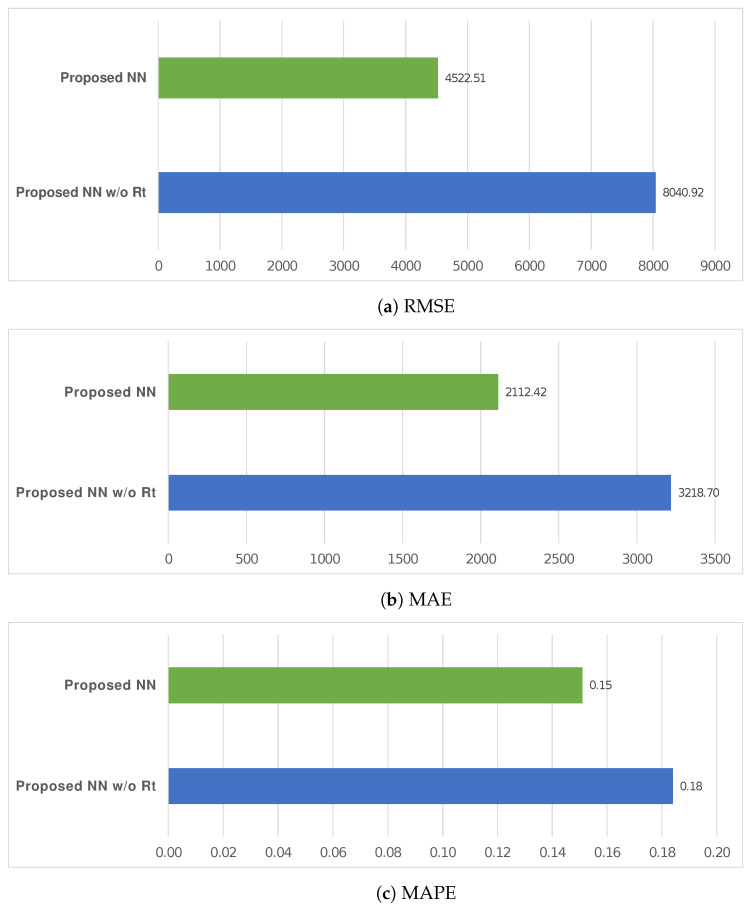
RMSE, MAE, and MAPE were used to compare the proposed NN and an NN with the same configuration but without the adjustment based on $R_{t}$. Values related to this plot are reported in Table 5.

In addition, RMSE, MAE, and MAPE were used to compare the proposed NN and an NN having the same configuration but without the adjustment based on $R_{t}$ (i.e., proposed NN and proposed NN without $R_{t}$, respectively), on the Italian dataset. In this case, we reported all KPIs so that both models were based on the same type of model and the comparison was possible, unlike the previous case. The effect of our approach improved all KPIs, as shown in Figure 6; Table 5 reports the related descriptive statistics.

We focused the tests on the Italian dataset, and by extending these ones also to the USA, France, the UK, and Sweden we demonstrated the evolvability and reusability of our solution for data based on time series. MAPE was calculated for the proposed NN with and without the correction computed by using the $R_{t}$ estimation (i.e., proposed NN and proposed NN without $R_{t}$, respectively), as well as for the other models based on DL (i.e., LSTM, Simple RNN, GRU). In addition, in this case, the forecasting was performed 10 times for each model and the resulting average value was reported (see Table 6).

**Figure 7:**
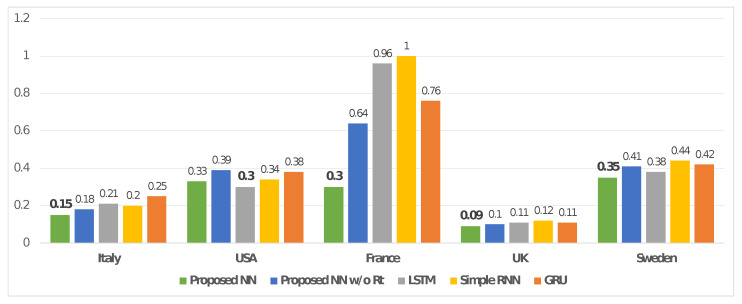
Figure shows a comparison between the proposed model with and without the correction computed by using the $R_{t}$ estimation (i.e., proposed NN and proposed NN w/o $R_{t}$, respectively), as well as from the test on the other models based on DL (i.e., LSTM, Simple RNN, GRU) and ARIMA. The comparison is based on MAPE, and it concerns all countries of interest: Italy, USA, France, UK, and Sweden. MAPE is oriented negatively (lower is better).

We summarized the MAPE provided by the presented tests on the DL models of interest (i.e., proposed NN, proposed NN without $R_{t}$, LSTM, Simple RNN, GRU) by plotting a bar chart. The latter is shown in Figure 7.

**Figure 8:**
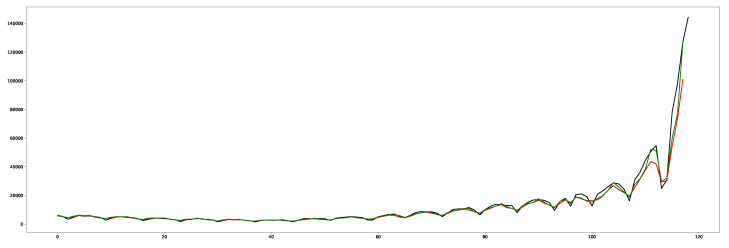
This plot shows a forecast related to the positive cases in Italy. The green line is related to the proposed solution that includes our adjustment based on $R_{t}$ estimation; the red line an NN based on a model built on the same configuration without $R_{t}$; the black line concerns the real trend for the period of interest. The X-axis reports the time points, while the Y-axis reports estimated positive cases.

For demonstration purposes only, Figure 8 shows a forecast related to the positive cases in Italy. It consists of the following information: the green line is related to the proposed solution that includes our adjustment based on $R_{t}$ estimation; the red line an NN based on a model built on the same configuration without $R_{t}$; the black line concerns the real trend for the period of interest.

In addition, we investigated the statistical significance related to the use of $R_{t}$ by performing a paired-sample *t*-test (or dependent-sample *t*-test) [65] before and after applying it to the proposed solution. Therefore, we considered the predicted time points with (i.e., after) and without (i.e., before) applying $R_{t}$: the green line and the red line, respectively, in Figure 8. Table 7 reports the results. Each time point was measured twice, resulting in pairs of observations.

## 5. Discussion

We built the time-series by processing the positive cases available within the dataset of the following countries: Italy, the USA, France, the UK, and Sweden. We focused on the Italian dataset, and extended the analysis to other mentioned countries. Each dataset was partitioned into two subsets for training (train set) and testing (test set), 80% and 20% respectively. After this first splitting, we kept aside the test set and we randomly chose 80% of the train set as the new train set, and the remaining (20%) was the validation set. The model was iteratively trained and validated on these sets. The train and validation sets were also used as entry points for $R_{t}$ estimation. Furthermore, the predicted values were evaluated on the data produced by the output layer of our model, iteratively. Finally, $R_{t}$ was applied as weight for each predicted value before providing the final output.

The KPIs (i.e., MAPE, MAE, RMSE) used for comparisons were oriented negatively: lower was better. On the Italian dataset, the results showed that our solution reduced MAPE by 28.44%, 39.36%, 22.96%, 17.93%, 28.10%, 24.50% compared to other ones with the same configuration but based on LSTM, GRU, Simple RNN, ARIMA (1,0,3), and ARIMA (7,2,4) models, or an NN without applying the $R_{t}$ as a corrective index (i.e., proposed NN without $R_{t}$). Furthermore, it reduced MAPE by 17.93%, MAE by 34.37%, and RMSE by 43.76% compared to the same model without the output adjustment performed through the $R_{t}$. Table 4 and Figure 5 report what has been described. Furthermore, Table 5 and Figure 6 show that our solution allowed a reduction of 17.93% in the MAPE, 34.37% in the MAE, and 43.76% in the RMSE compared to an NN that did not include the $R_{t}$ estimation as a corrective index.

In addition, the tests performed on the other countries were consistent with what was described above. According to Table 6 and the related plot in Figure 7, a comparison between our solution and other ones provided the following reduction in the MAPE indicator for the USA, France, the UK, and Sweden:

- vs. proposed without $R_{t}$: 5.38%, 53.13%, 10.00%, and 14.63%;
- vs. LSTM: −10.00%, 68.75%, 18.18%, and 7.89%;
- vs. Simple RNN: 2.94%, 70.00%, 25.00%, and 20.45%;
- vs. GRU: 13.16%, 60.53%, 18.18%, and 16.67%,

It is important to underline that only the comparison on the USA dataset between our solution and the model based on LSTM provided a better result for the second model (−10.00% for LSTM). However, the proposed solution allowed an average MAPE reduction of 5.37%, 63.10%, 17.84%, and 14.91% on the datasets related to the USA, France, the UK, and Sweden, respectively.

Italy was subjected to emergency improvements that introduced restrictions for preventing the spread of COVID-19, as well as France and the UK. On the contrary, the USA and Sweden introduced a more permissive policy (no or low restrictions). Overall, the described results identified the proposed solution as the most appropriate in our tests, especially for time series affected by influences over the time (e.g., lockdowns and limitations).

Furthermore, the statistical analysis related to the use of $R_{t}$ applied to the proposed solution (see Table 7) indicated a statistical significance (*p*-value of 0.01). This analysis was related to the forecast in Figure 8.

## 6. Conclusions

This paper presented a DL algorithm for the analysis and forecasting of SARS-CoV-2 (COVID-19) epidemic trends by combining an NN with $R_{t}$ estimation. In the proposed solution, the data produced by the output layer of the NN were weighted on $R_{t}$ estimation. The $R_{t}$ was included as a multiplying factor at the end of the output layer, making sure that the estimated contagion rate could afflict the original output by weighting it.

We applied the proposed solution to the datasets of Italy, the USA, France, the UK, and Sweden, by focusing the attention on the Italian dataset. Results showed that our solution allowed a reduction in MAPE of 28.44%, 39.36%, 22.96%, 17.93%, 28.10%, 24.50% compared to other ones with the same configuration but based on LSTM, GRU, RNN, ARIMA (1,0,3), and ARIMA (7,2,4) models, or an NN without applying the $R_{t}$ as a corrective index. It also allowed a reduction of 17.93% in MAPE, 34.37% in MAE, and 43.76% in RMSE compared to an NN that did not include the $R_{t}$ estimation as a corrective index. Furthermore, it allowed an average MAPE reduction of 5.37%, 63.10%, 17.84%, and 14.91% on the datasets related to the USA, France, the UK, and Sweden, respectively.

Future work could affect the tracking of SARS-CoV-2 variants by evaluations that take into account multiple rates of contagiousness, as well as including sets of information both for personal and clinical data (e.g., clustering data for ages and comorbidities, susceptible patients, and statistics on mobility).

## Key Points

- We combined an NN with the estimation of time-varying reproduction numbers during epidemics (i.e., using $R_{t}$ as a corrective index).
- We developed a solution that was able to handle the frequent restrictions adopted by a country (e.g., lockdowns and limitations) that could destabilize the evolution of a time series.
- We provided an effective methodology to forecast COVID-19 epidemic trends on a dataset consisting of a limited amount of information (e.g., Italy).

## Figures and Tables

**Table 1:** We obtained the information related to positive cases in Italy, the USA, France, the UK, and Sweden, through the “Our World in Data COVID-19 Cases” dataset. For the mentioned countries, it aggregated data from the sources accessed on 11 January 2022, and reported in this table. The related time series were built based on the number of daily new positive cases and whose timestamp was within the same date range (24 February 2020–11 January 2022).

Country	Organization	Data Source
Italy	Italian Civil Protection Department	github.com/pcm-dpc/COVID-19
USA	Center for Systems Science and Engineering, Johns Hopkins University	systems.jhu.edu
France	French Ministry of Solidarity and Health and Public Health	data.gouv.fr/fr/datasets
UK	Government of the UK	coronavirus.data.gov.uk
Sweden	The Swedish Public Health Agency	experience.arcgis.com

**Table 2:** The descriptive statistics both for new cases and total cases, in Italy, the USA, France, the UK, and Sweden. Date range: 24 February 2020–11 January 2022 (*N* is the number of time points for the time series, which is the number of evaluated days).

		N	Mean	Std. Deviation	Std. Error	95% Confidence Interval for Mean		Minimum	Maximum
						Lower Bound	Upper Bound		
**Total Cases**	**Italy**	688	2,496,779.68	2,007,837.429	76,548.084	2,346,483.41	2,647,075.95	229	7,774,863
	**USA**	688	22,722,404.82	17,313,142.593	660,057.373	21,426,432.95	24,018,376.69	16	62,588,935
	**France**	688	3,581,853.14	3,034,266.023	115,680.308	3,354,723.75	3,808,982.52	12	12,620,080
	**UK**	688	3,729,463.11	3,563,954.756	135,874.501	3,462,683.98	3,996,242.24	30	14,766,757
	**Sweden**	688	588,621.05	487,097.434	18,570.416	552,159.46	625,082.63	1	1,487,291
**New Cases**	**Italy**	688	11,317.11	22,531.009	859.612	9629.33	13,004.90	74	220,519
	**USA**	688	90,972.27	115,946.169	4420.406	82,293.14	99,651.39	0	1,383,898
	**France**	688	19,238.68	38,431.340	1475.947	16,340.70	22,136.67	0	368,379
	**UK**	688	21,536.37	29,864.863	1140.246	19,297.58	23,775.17	2	219,290
	**Sweden**	688	2161.76	5199.062	198.212	1772.58	2550.93	0	70,641

**Table 3:** The descriptive statistics related to the study of the $R_{t}$ index in the countries of interest.

	N	Mean	Std. Deviation	Minimum	25%	50%	75%	Maximum
**Italy**	688	1.05	0.23	0.70	0.89	1.00	1.14	2.98
**USA**	688	1.08	0.33	0.74	0.91	1.03	1.13	3.74
**France**	688	1.32	2.99	0	0.91	1.06	1.24	56.87
**UK**	688	1.07	0.32	0.59	0.90	1.04	1.18	5.95
**Sweden**	688	1.11	0.622	0.02	0.93	1.06	1.20	10.04

**Table 4:** The MAPE indicator was used to compare the proposed NN and the following models: LSTM, GRU, Simple RNN, ARIMA. It was oriented negatively (lower was better). Dataset: Italy.

					95% Confidence Interval for Mean			
	N	Mean	Std. Deviation	Std. Error	Lower Bound	Upper Bound	Minimum	Maximum
**LSTM**	10	0.2110	0.03281	0.01038	0.1875	0.2345	0.18	0.28
**GRU**	10	0.2490	0.04533	0.01433	0.2166	0.2814	0.20	0.31
**Simple RNN**	10	0.1960	0.05125	0.01621	0.1593	0.2327	0.16	0.33
**Proposed NN w/o** $R_{t}$	10	0.1840	0.01713	0.00542	0.1717	0.1963	0.16	0.21
**Proposed NN**	10	0.1510	0.01101	0.00348	0.1431	0.1589	0.14	0.17

**Table 5:** RMSE, MAE, and MAPE were used to compare the proposed NN and an NN with the same configuration but without the adjustment based on $R_{t}$. It was oriented negatively (lower was better). The figures show the proposed solution in green, and the other models are in blue. Furthermore, we reported our solution by excluding the use of $R_{t}$ in yellow to demonstrate the benefits of using the latter. Dataset: Italy.

						95% Confidence Interval for Mean			
		N	Mean	Std. Deviation	Std. Error	Lower Bound	Upper Bound	Minimum	Maximum
**RMSE**									
	**Proposed NN w/o** $R_{t}$	10	8040.9180	1181.94026	373.76233	7195.4089	8886.4271	6224.14	9763.09
	**Proposed NN**	10	4522.5080	2645.23581	836.49701	2630.2203	6414.7957	1407.01	7123.64
**MAE**									
	**Proposed NN w/o** $R_{t}$	10	3218.7000	922.85482	291.83232	2558.5294	3878.8706	2122.75	4813.67
	**Proposed NN**	10	2112.4220	1169.72987	369.90106	1275.6477	2949.1963	918.19	4010.12
**MAPE**									
	**Proposed NN w/o** $R_{t}$	10	0.1840	0.01713	0.00542	0.1717	0.1963	0.16	0.21
	**Proposed NN**	10	0.1510	0.01101	0.00348	0.1431	0.1589	0.14	0.17

**Table 6:** MAPE was reported for the proposed model with and without the correction computed by using the $R_{t}$ estimation, as well as for the other models based on DL (i.e., LSTM, Simple RNN, GRU). The forecasting was performed 10 times for each model and the resulting average value was reported. MAPE was oriented negatively (lower was better).

	Proposed NN	Proposed NN w/o $R_{t}$	LSTM	Simple RNN	GRU
**USA**	0.33	0.39	0.30	0.34	0.38
**France**	0.30	0.64	0.96	1.00	0.76
**UK**	0.09	0.10	0.11	0.12	0.11
**Sweden**	0.35	0.41	0.38	0.44	0.42

**Table 7:** The paired-sample *t*-test before and after applying the $R_{t}$ adjustment to the proposed solution. Dataset: Italy.

Mean	Std. Deviation	Std. Error Mean	95% Confidence Interval of the Difference		*p*-Value
			Lower	Upper	
−646.23	2690.34	247.67	−1136.72	−155.74	0.01
